# abLIM1 constructs non-erythroid cortical actin networks to prevent mechanical tension-induced blebbing

**DOI:** 10.1038/s41421-018-0040-3

**Published:** 2018-07-24

**Authors:** Guoqing Li, Shan Huang, Sen Yang, Jiabin Wang, Jingli Cao, Daniel M. Czajkowsky, Zhifeng Shao, Xueliang Zhu

**Affiliations:** 1 State Key Laboratory of Cell Biology, CAS Center for Excellence in Molecular Cell Science, Shanghai Institute of Biochemistry and Cell Biology, Chinese Academy of Sciences, University of Chinese Academy of Sciences, 320 Yueyang Road, Shanghai, 200031 China; 2 Shanghai Center for Systems Biomedicine, Key Laboratory of Systems Biomedicine (Ministry of Education), Shanghai Jiao Tong University, 800 Dongchuan Road, Shanghai, 200240 China; 3 Bio-ID Center, School of Biomedical Engineering, Shanghai Jiao Tong University, 800 Dongchuan Road, Shanghai, 200240 China

## Abstract

The cell cortex is a layer of cytoskeletal networks underneath the plasma membrane, formed by filamentous actin (F-actin) and cortex proteins including spectrin, adducin, and myosin. It provides cells with proper stiffness, elasticity, and surface tension to allow morphogenesis, division, and migration. Although its architecture and formation have been widely studied in red blood cells, they are poorly understood in non-erythrocytes due to structural complexity and versatile functions. In this study, we identify the actin-binding protein abLIM1 as a novel non-erythroid cell-specific cortex organizer. Endogenous abLIM1 colocalized with cortical βII spectrin but upon overexpression redistributed to thick cortical actin bundles. abLIM1 associated with major cortex proteins such as spectrins and adducin in vivo. Depletion of abLIM1 by RNAi induced prominent blebbing during membrane protrusions of spreading or migrating RPE1 cells and impaired migration efficiency. Reducing cortical tensions by culturing the cells to confluency or inhibiting myosin activity repressed the blebbing phenotype. abLIM1-depleted RPE1 or U2OS cells lacked the dense interwoven cortical actin meshwork observed in control cells but were abundant in long cortical actin bundles along the long axis of the cells. In-vitro assays indicated that abLIM1 was able to crosslink and bundle F-actin to induce dense F-actin network formation. Therefore, abLIM1 governs the formation of dense interconnected cortical actin meshwork in non-erythroid cells to prevent mechanical tension-induced blebbing during cellular activities such as spreading and migration.

## Introduction

The cell cortex is a thin layer of actin network underneath and anchored to the plasma membrane, ranging from 50 nm to 2 μm in thickness. It is important for shape, division, migration, and morphogenesis of animal cells. It also modulates membrane microdomains and contributes to transmembrane processes such as endocytosis and exocytosis^1–8^.

The most studied cell cortex is that of red blood cells. The erythroid cortex is a polygonal meshwork composed of αΙ and βΙ spectrin tetramers cross-linked at nodes by short filamentous actin (F-actin) and other cortex proteins such as adducin, ankyrin, dematin, and tropomyosin^5, 7, 9^. It is pinned to the plasma membrane through associations with phosphatidylinositol lipids and transmembrane proteins^7, 9^. Mutations in the cortex proteins cause defected erythroid morphology and function^9^.

By contrast, non-erythroid cortexes are mostly irregular and dynamic in structure and are mainly composed of F-actin networks^10–13^. Only neurons have recently been found to contain ordered cortical actin structures along their neurites, in which short actin filaments are proposed to form rings of 180 to 190-nm periodicity interspaced laterally by spectrin tetramers^14–16^. Although non-erythrocytes use different spectrin paralogs (such as αII and βII spectrins), they appear to share other cortical cytoskeleton components with erythrocytes^5, 7, 9, 14^. How a similar set of cortical proteins can organize such diverse cytoskeletal networks in different cellular context is not known. One possibility is that unidentified actin regulators contribute to the construction of the non-erythroid cortexes. This, however, is not documented to date.

Vertebrate abLIM1-3 are poorly studied actin-binding proteins. Their N-terminal halves contain four zinc-binding LIM domains, whereas their C-terminal halves are entirely homologous to dematin (see Supplementary Fig. 1)^17–21^. abLIM1-3 appear to show both overlapping and distinct expressing patterns in different tissues or cells^17, 20, 21^. abLIM1 and abLIM2 localize to the lateral boundary of the sarcomere, or the z-discs, of striated muscles^17, 20, 22^. Consistent with their actin-binding properties, the abLIM proteins display stress fiber-like localizations upon overexpression and are important for cell migration^17, 20, 23^. Furthermore, depletion of abLIM1 reduces the number of stress fibers in NIH3T3 cells, whereas its overexpression increases cellular F-actin^24, 25^.

We have previously found that depletion of abLIM1 or abLIM3 by RNAi markedly promotes ciliogenesis in the presence of serum in cultured cells by influencing actin dynamics^23^. In this report, we identify abLIM1 as a novel component of the non-erythroid cortex that is critical for the formation of cortical F-actin networks and proper plasma membrane-cell cortex attachment under mechanical tension.

## Results

### abLIM1 is a non-erythroid cortex protein

**Fig. 1:**
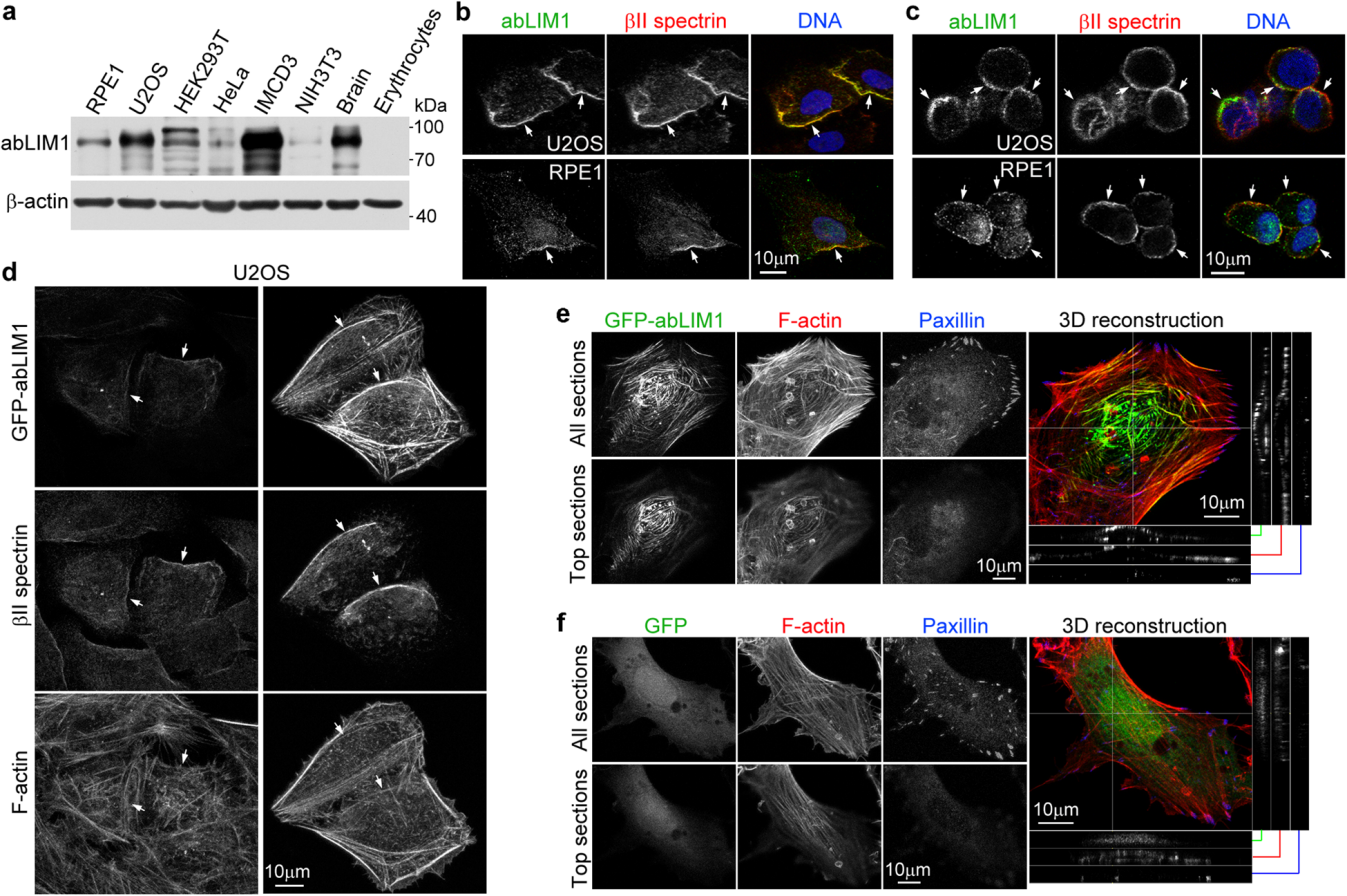
abLIM1 is a non-erythroid cortex protein. **a** Expression of abLIM1 in different cells or mouse tissues. β-actin served as loading control. **b**, **c** abLIM1 was a cell cortex protein. Intact U2OS or RPE1 cells (**b**) or the cells treated with EDTA to acquire a spherical morphology (**c**) were subjected to immunostaining and confocal microscopy. A single optical section is shown for the cells in (**c**). βII spectrin served as cell cortex marker. Arrows point to typical regions positive for both proteins. Nuclear DNA was visualized by DAPI. **d** Subcellular localization of GFP-abLIM1 in U2OS cells. Arrows indicate regions apparently positive for GFP-abLIM1, βII spectrin, and F-actin. Note that highly expressed GFP-abLIM1 displayed colocalization with actin bundles. **e** Overexpressed GFP-abLIM1 colocalized with cortical actin bundles. Shown are confocal micrographs of a representative RPE1 cell. The two dimensional (2D) images were projected from all optical sections or just Paxillin-free top sections. The 3D reconstructed image is shown as the top view, accompanied with side views of the indicated positions. Paxillin signals mark the bottom side of the cell. **f** GFP failed to show associations with F-actin in RPE1 cells. Note that the cell is also abundant in cortical actin bundles

abLIM1 showed varying expression levels in cultured cells and mouse tissues but was undetectable in red blood cells (Fig. 1a)^17^. Immunostaining revealed that it was highly enriched at cell edges in RPE1 and U2OS cells, where its immunofluorescent signals colocalized with those of βII spectrin (Fig. 1b), a cell cortex marker^5, 7^. To validate the antibody specificity, we pre-incubated the anti-abLIM1 antibody with purified polyhistidine (His)-tagged human abLIM1, abLIM3, or GFP and found that only the pre-incubation with His-abLIM1 abolished the cortical immunofluorescent signals (Supplementary Fig. 2a,b). Depletion of abLIM1 using abL1-i1, a previously described siRNA^23^, also abolished the signals (Supplementary Fig. 2c,d). Furthermore, when the cells were partially detached from the substratum to become roundup, abLIM1 was seen clearly at the cortex, being more punctate than βII spectrin (Fig. 1c). Thus, abLIM1 is a cell cortex protein specifically in non-erythrocytes.

Interestingly, upon overexpression abLIM1-3 exhibit prominent colocalization with actin bundles^20, 23^. We found that exogenous abLIM1 at low expression levels tended to assume endogenous abLIM1-like distributions but at high expression levels expanded to actin bundles in addition to the colocalization with βII spectrin (Fig. 1d and Supplementary Fig. 2e). To clarify the identity of these bundles, we used paxillin, a focal adhesion protein, to mark the bottom side of the cells^26, 27^ and analyzed z-stack images of GFP-abLIM1-expressing RPE1 cells. We found that a portion of the F-actin bundles and their associated GFP-abLIM1 were actually distributed along the top surface of the cells (Fig. 1e), indicating that they belong to cortical actin. Such cortical F-actin bundles also existed in intact cells or cells overexpressing GFP (Fig. 1f). Therefore, abLIM1 is absent in spectrin-free cortical actin bundles unless being overexpressed.

### Depletion of abLIM1 results in blebbing during cell spreading

**Fig. 2:**
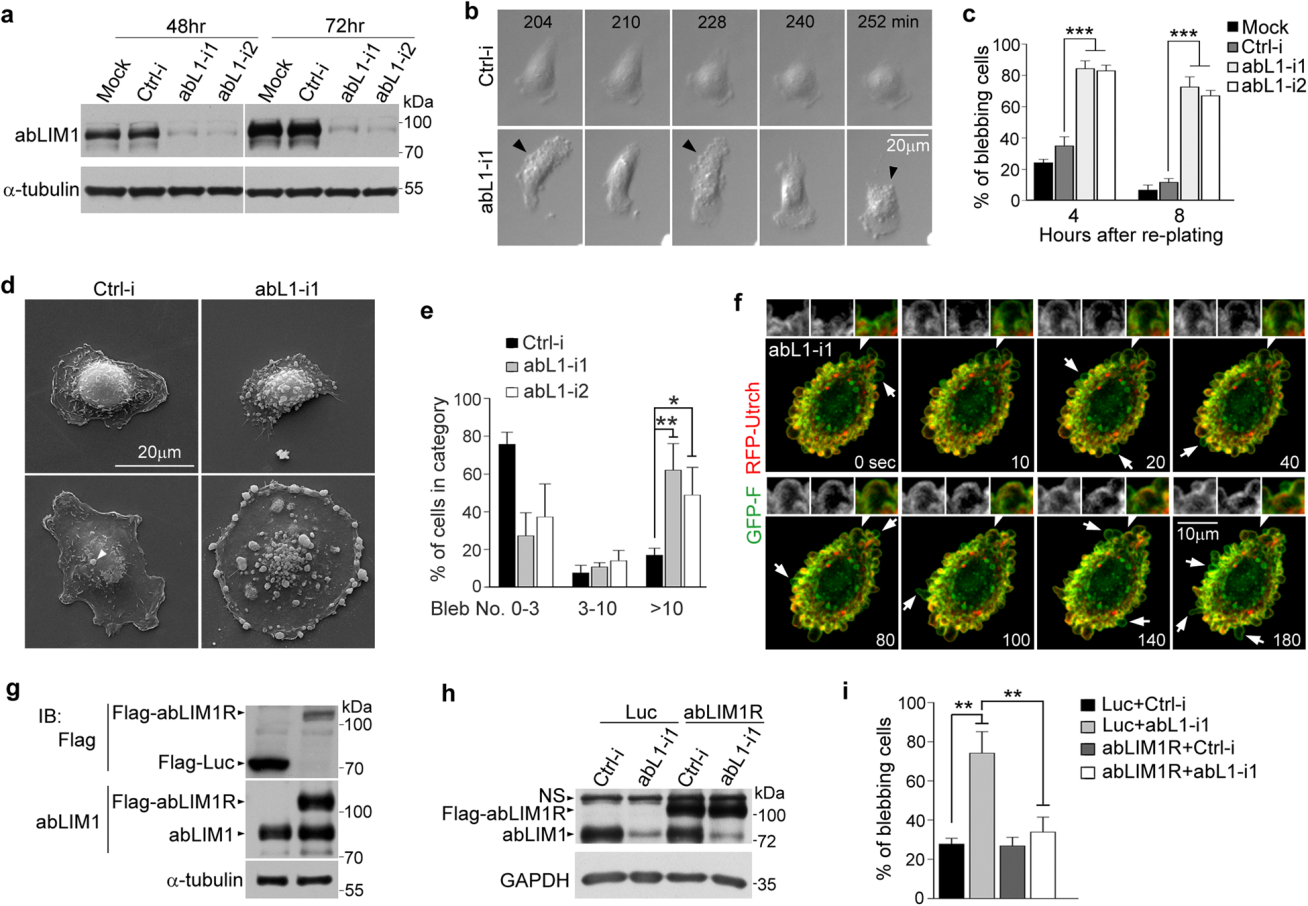
Depletion of abLIM1 results in blebbing during cell spreading. Pooled data are presented as mean ± s.d. Student’s *t* test: *P < 0.05; **P < 0.01; ***P < 0.001. **a** Efficient knockdown of abLIM1 by two different siRNAs (abL1-i1 and -i2) in RPE1 cells. Ctrl-i is a control siRNA. α-tubulin served as loading control. **b** abLIM1-depleted cells displayed repetitive protrusion-blebbing-retraction cycles during spreading and migration. The images were from Supplementary Video 1. RPE1 cells transfected for 48 h were re-plated at 10% confluency and imaged for 10 h at 6-min intervals. The arrowheads indicate the blebbing stage of the cell. **c** Quantification results from three independent experiments. Micrographs at 4 and 8 h of the live imaging as in (**b**) were used for the analysis. At least 60 cells were scored in each experiment and condition. **d** Scanning EM images for cells showing different extent of spreading. RPE1 cells transfected for 48 h were re-plated at 10% confluency for 4 h and processed for the EM. The arrowhead indicates a bleb in control cell. **e** Quantification results from three independent experiments as in (**d**). At least 60 cells were scored in each experiment and condition. **f** Representative frames of a typical blebbing cell. RPE1 cells stably expressing GFP-F and RFP-Utrch to label plasma membrane and F-actin, respectively, were transfected for 48 h with abL1-i1 and re-plated at 10% confluency. After 4 h of culture, the cells were imaged at 10-sec intervals. The frames were from Supplementary Video 2. The magnified (2×) views show a typical blebbing process. Other nascent blebs are indicated by arrows. **g** Expression levels of RNAi-insensitive abLIM1R and luciferase (Luc) in stable cells for rescue experiments. RPE1 cells infected with lentivirus were sorted out by FACS, based on GFP expressed from the lentiviral constructs through an internal ribosome entry site (IRES), and maintained as stable cell lines. α-tubulin served as loading control. **h** Depletion of endogenous abLIM1 by RNAi in the stable RPE1 cells. GAPDH served as loading control. NS, non-specific band. **i** abLIM1R repressed the blebbing of the abLIM1-depleted cells. The stable RPE1 cells transfected for 48 h were re-plated at ~10% confluency. The spreading cells were then imaged for at least 240 min at 6-min intervals. The statistical results, from three independent experiments, were obtained using the micrographs at 240 min. Approximately 100 cells were scored in each experiment and condition

To understand whether abLIM1 has a role in the cortical actin network, we depleted abLIM1 in RPE1 cells (Fig. 2a) and examined spreading behaviors of the cells after re-plating. Compared to control cells that were either mock-transfected or transfected with a control siRNA, ctrl-i^23^, those transfected with abL1-i1 or abL1-i2 tended to manifest numerous tiny and dynamic puffs all over the cells (Fig. 2b,c and Supplementary Video 1). Some of the puffs emerged from the cell edge (Fig. 2b and Supplementary Video 1), suggesting that they are membrane blebs^4, 28, 29^. We found that massive blebs suddenly broke out during cell protrusion, resulting in premature retraction of the leading edge, during which process the blebs gradually reduced in number or even vanished. Accordingly, the cells underwent repetitive spreading-blebbing-retraction cycles. Control cells, however, displayed much less extent of blebbing (Fig. 2b, c and Supplementary Video 1). Scanning electron microscopy (EM) revealed that the cells treated with abL1-i1 or -i2 were abundant in membrane blebs of varying sizes (Fig. 2d, e). 49 ± 15% or more of the cells contained >10 blebs of varying sizes per cell. By contrast, the value was 17 ± 4% in the ctrl-i treated populations (Fig. 2d, e). When GFP-F and RFP-Utrch were stably expressed to simultaneously label the plasma membrane and F-actin, respectively (Fig. 2f and Supplementary Fig. 3)^30–32^, the cells treated with abL1-i1 displayed typical blebbing behaviors. Initially a patch of plasma membrane free of RFP-Utrch rapidly puffed up. F-actin, as indicated by RFP-Utrch, was then progressively assembled at the membrane domain as the blebs grew. After reaching the maximal RFP-Utrch intensity, the blebs started to retract (Fig. 2f and Supplementary Video 2)^4, 12^. Thus the depletion of abLIM1 leads to membrane blebbing during cell spreading.

To rule out the off-target effect of the RNAi experiments, we created an RNAi-resistant isoform of abLIM1 (hereafter termed abLIM1R) by mutating the target sequence of abL1-i1 without altering the coding amino acids. We established an RPE1 cell line stably expressing Flag-HA-abLIM1R to levels close to those of the endogenous abLIM1 (Fig. 2g). Depleting endogenous abLIM1 from the stable RPE1 cells by RNAi no longer triggered blebbing during cell spreading (Fig. 2h, i). By contrast, the RNAi-induced blebbing was still prominent in RPE1 cells stably expressing Flag-HA-luciferase (Fig. 2g-i). abL1-i1 is thus specific to abLIM1.

### Depletion of abLIM1 impairs cell migration efficiency by causing blebbing

**Fig. 3:**
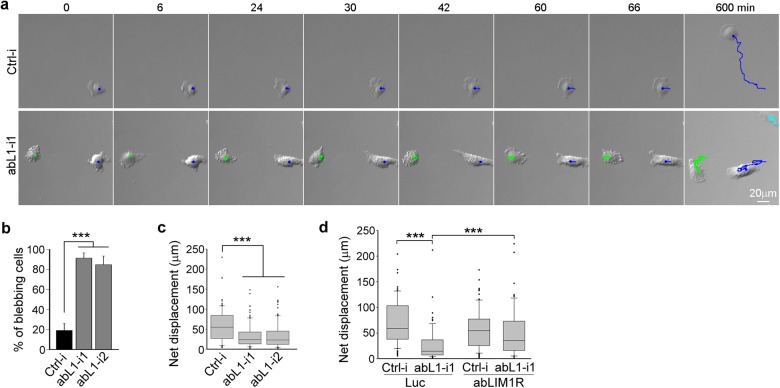
Depletion of abLIM1 results in blebbing during free cell migration. **a** Representative frames for the migrating cells in Supplementary Video 3. RPE1 cells transfected with siRNA for 36 h were re-plated at 10% confluency. 12 h after the re-plating, the cells were imaged for 10 h at 6-min intervals. Migration trajectories (colored lines) of the cells over 600 min are also plotted. Please note the repetitive spreading-blebbing-retraction cycles of the abL1-i1-treated cell to the left. **b** Statistical results on blebbing cells from three independent experiments. 30 to 40 cells imaged as in (**a**) were scored in each experiment and condition. Cells repeatedly displayed repeated blebbing were counted as blebbing cells. Data are presented as mean ± s.d. Student’s *t* test: *** P < 0.001. **c** abLIM1-depleted RPE1 cells exhibited poor migration efficiency. Net displacement in 10 h was measured for free-migrating cells imaged as in (**a**). The box-plots were from three independent experiments. 30-40 cells were scored in each experiment and condition. The bottom and top of the box represent the 25th and 75th percentiles, respectively; the band is the median; and the ends of the whiskers indicate the 90th and 10th percentiles of the data. The dots are outliers. Student’s *t* test: ***P < 0.001. **d** abLIM1R restored the free migration defects of abLIM1-depleted cells. RPE1 cells stably expressing Flag-HA-abLIM1 or -luciferase were transfected with the indicated siRNAs for 36 h and re-plated at 10% confluency. After culturing for additional 12 or 24 h, the cells were imaged for 10 h at 6-min intervals. Net displacements were scored for free migrating cells in three independent experiments. 24 cells were quantified in each experiment and condition. Student’s *t* test: ***P < 0.001

We have previously shown that abLIM1 is required for efficient directional cell migration in wound healing assays^23^. As cell migration also involves membrane protrusion, we examined migrating behaviors of abLIM1-depleted RPE1 cells. To avoid influences of neighboring cells, we seeded the cells sparsely for at least 12 h and monitored their free migrations through live imaging. We found that, similar to the spreading cells (Fig. 2b, c), these migrating cells also displayed spreading-blebbing-retraction cycles: blebbing emerged all over the cells during plasma membrane protrusions, followed by membrane retraction (Fig. 3a, b and Supplementary Video 3). Accordingly, the cells tended to oscillate around their initial positions and exhibit poor migration efficiency (Fig. 3a and Supplementary Video 3). When the net displacement in 10 h was measured to reflect the ability of directional migration, the median for the control cells was 55.1 μm (Fig. 3c). By contrast, the value was only 23.8 and 23.2 μm for the cells treated with abL1-i1 or abL1-i2, respectively (Fig. 3c).

To exclude off-target effect, we transfected the stable RPE1 cells expressing Flag-HA-abLIM1R or -luciferase with ctrl-i or abL1-i1 (Fig. 2g, h) and examined their migration abilities. Upon the depletion of the endogenous abLIM1, the cells expressing abLIM1R indeed displayed markedly increased net displacements as compared to those expressing luciferase (Fig. 3d). Therefore, abLIM1-depleted cells lack persistent directionality in free migration due to protrusion-induced blebbing.

### abLIM1-induced cortical actin antagonizes mechanical tension-induced blebbing

**Fig. 4:**
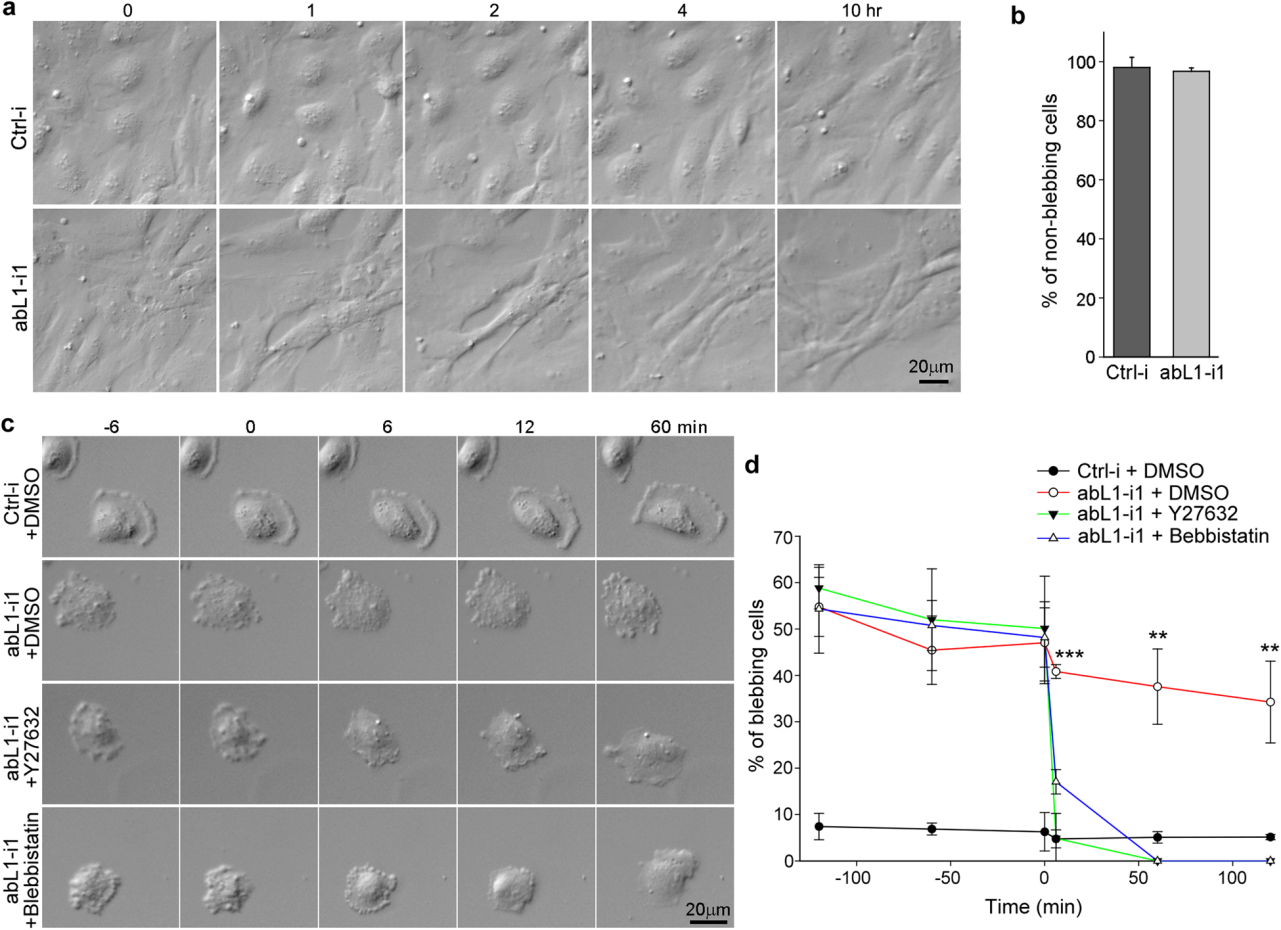
Reducing cortical tension suppresses abLIM1 depletion-induced blebbing. **a**, **b** The blebbing was suppressed in confluent cells. RPE1 cells transfected with siRNA for 36 h were re-plated at high density. After 12 h, the cells were imaged for 10 h at 6-min intervals and frames at the indicated time are shown. The statistical results on non-blebbing cells (**b**) were from three independent experiments. 50 cells were scored in each experiment and condition. Cells that showed visible blebbing in more than 10 frames out of the total 101 frames were considered as blebbing cells. Data are presented as mean ± s.d. **c**, **d** The blebbing was suppressed by inhibiting myosin II activity. RPE1 cells transfected for 36 h were re-plated at 10% confluency. 12 h after the re-plating, the cells were imaged at 6-min intervals. DMSO, blebbistatin, or Y27632 was added to the culture medium during the imaging (marked as 0 min) (**c**). Blebbing cells were quantified at 60-min intervals relative to the 0-min point in three independent experiments (**d**). At least 80 cells were scored in each experiment and condition

To clarify whether the blebbing of abLIM1-depleted cells was due to mechanical tension, we examined confluent RPE1 cells, which lack the membrane protrusion-induced tension. Indeed, blebbing was no longer observed in confluent cells upon abLIM1 depletion when examined by live imaging (Fig. 4a, b).

As repressing myosin II activity can reduce cortical tension and subsequently repress tension-induced bleb formation^29, 33, 34^, we treated abLIM1-depleted RPE1 cells with blebbistatin, a myosin II inhibitor, or Y27632, which inhibits the myosin II-activator Rho-associated protein kinase (ROCK)^29^. Immediately following the addition of the drugs, the blebbing effect of the abLIM1-depleted RPE1 cells was markedly suppressed (Fig. 4c, d). The depletion of abLIM1, however, did not augment myosin II activities when the phosphorylation levels of myosin light chain 2 (MLC2, also called RLC)^35, 36^ were assessed (Supplementary Fig. 4). Phosphorylation levels of the ezrin, radixin, and moesin proteins (ERMs), important anchors of cortical actin to the plasma membrane^37^, were grossly unaffected as well (Supplementary Fig. 4). Taken together, we conclude that the cortical actin networks assembled by abLIM1 function to counteract mechanical tension during cellular activities such as membrane protrusion.

### abLIM1 is essential for the formation of dense interwoven cortical meshwork

**Fig. 5:**
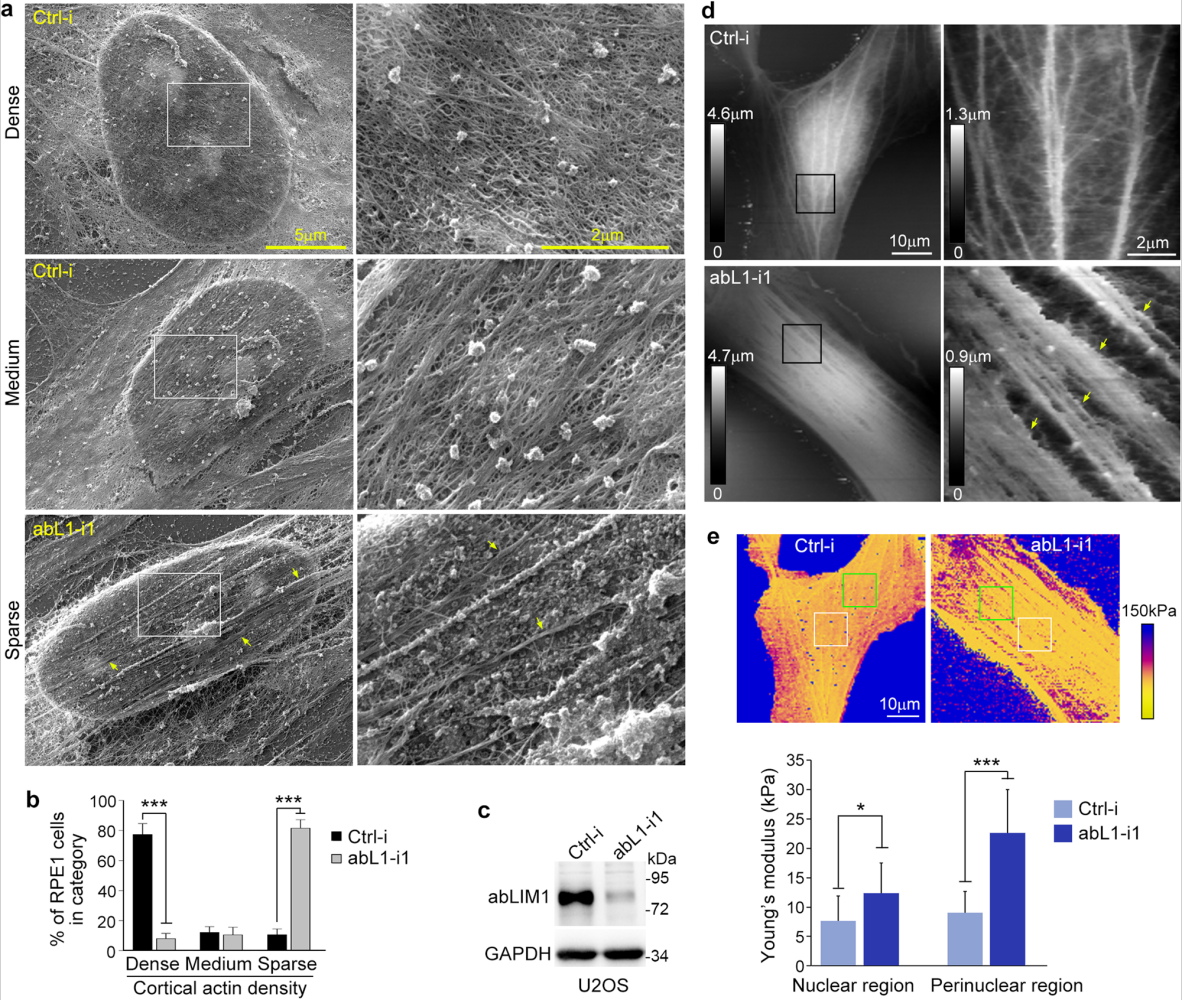
abLIM1 depletion impairs the interwoven cortical actin meshwork. Pooled data are presented as mean ± s.d. Student’s *t* test: *P < 0.05; ***P < 0.001. **a** Typical scanning EM images to show different (dense, medium, or sparse) cortical actin density. RPE1 cells transfected for 48 h were treated with Triton X-100 to remove the plasma membranes for scanning EM. Cells close to confluency were used to reduce influences of blebbing or membrane protrusion on cortical actin morphology. The framed areas were magnified to show details. Arrows indicate long cortical actin fibers in the abLIM1-depleted cell. **b** Quantification results from three independent experiments, based on the criteria in (**a**). At least 20 cells were scored in each experiment and condition. **c** abL1-i1 efficiently depleted abLIM1 in U2OS cells. GAPDH served as loading control. **d** AFM images. Living U2OS cells transfected for 72 h were imaged with the Peak Force mode of AFM. The framed regions were scanned at higher resolutions. The spectrum indicates height information. Arrows indicate long cortical actin fibers in the abLIM1-depleted cell. Note that the finest structures of the cortex as seen in (**a**) were not resolved because the radius of the AFM probe was 10 nm. **e** Increased cortical stiffness in the abLIM1-depleted U2OS cells. Young’s modulus maps for the cells in (**d**) are shown. The average Young’s modulus in a 10 μm × 10 μm region over (white frames) or beside (green frames) the nucleus was quantified. 10 cells from three independent experiments were analyzed in each group

To understand why the abLIM1-depleted cells are prone to blebbing, we visualized cortical actin with scanning EM. We treated RPE1 cells transfected with ctrl-i or abL1-i1 for 48 h with 1% Triton X-100 to remove plasma membranes and soluble biomolecules prior to fixation, as described previously^38^. The control RPE1 cells mostly displayed a dense cortical meshwork of interwoven filaments and their bundles (Fig. 5a, b). By contrast, the majority of abLIM1-depleted cells contained sparse cortical actin filaments (Fig. 5a, b). Concomitantly, bundles along the long axis of the cells became prominent (Fig. 5a).

To corroborate the EM results, we performed atomic force microscopy (AFM) to directly image the cortex in living cells^39^. As RPE1 cells often did not hold tightly enough on the substratum during the tip-scan imaging, we used U2OS cells instead (Fig. 5c). In the control cells, thick cortical fibers were found to branch hierarchically into numerous thinner interconnecting fibers to form intricate networks (Fig. 5d). By sharp contrast, the abLIM1-depleted cells were only abundant in arrays of thick fibers along the long axis. High resolution scan revealed that these cells clearly lacked the intricate fiber structures observed in the control cells (Fig. 5d). Accordingly, cortical stiffness in the abLIM1-depleted cells increased by 60% at nuclear regions and 151% at perinuclear regions as compared to the control cells (Fig. 5e).

To clarify whether the depletion of abLIM1 affected the thickness of cortical actin, we treated U2OS cells with EDTA to allow them to round up and acquired images by Stimulated Emission Depletion (STED) microscopy (Supplementary Fig. 5a), which can achieve a resolution higher than 100 nm at the x–y plane^40^. Cortical actin thickness was measured as 214.0 ± 15.6 nm for control cells and 216.4 ± 17.9 nm for abLIM1-depleted cells (Supplementary Fig. 5b, c).

Taken together, we conclude that depletion of abLIM1 altered structural organization of cortical actin by impairing its interwoven meshwork but enhancing thick long fibers.

### abLIM1 crosslinks and bundles F-actin into dense meshwork in vitro

**Fig. 6:**
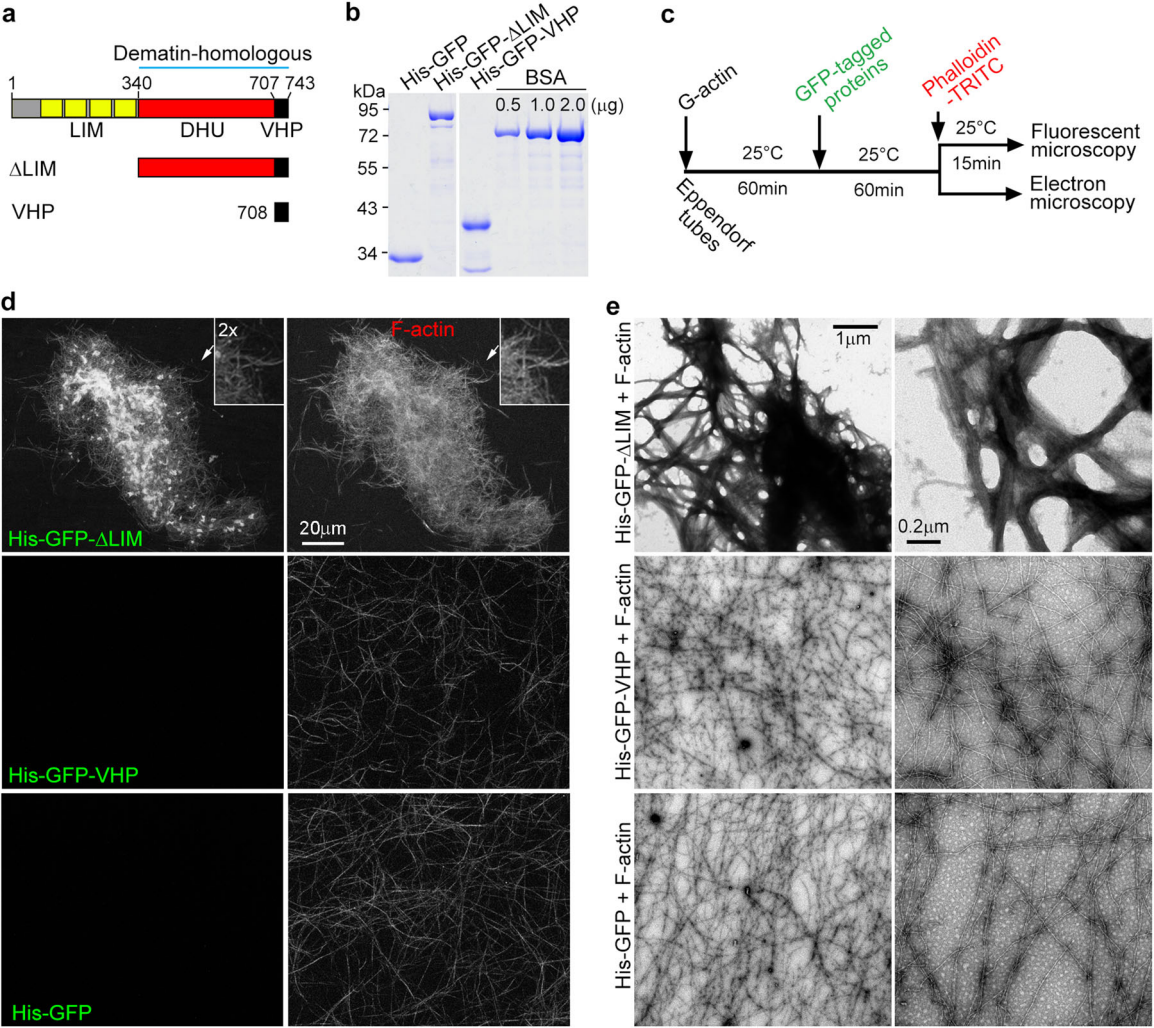
abLIM1 binds to and organizes F-actin into dense networks in vitro. **a** Diagrams of human abLIM1 and its mutants used for in vitro assays. LIM, LIM motifs; DHU, dematin-homologous unfolded region; VHP, villin headpiece domain. **b** Proteins expressed and purified from *E. coli*. Bovine serum albumin (BSA) was loaded for quantification. All the lanes were from the same gel. **c** Experiment scheme. See Methods for details. **d** ΔLIM induced F-actin network formation by binding to F-actin. Note that the VHP domain alone neither bound to F-actin nor led to actin network formation. **e** Negative staining EM indicated that ΔLIM crosslinked and bundled F-actin

Next we examined how abLIM1 could affect F-actin through in vitro assays. Full-length abLIM1 has been documented to be prone to degradation when expressed in *E. coli* but a short isoform (abLIM-s) containing only the dematin-homologous region can be expressed and shown to co-sediment with F-actin^17^. We expressed His or GST-tagged abLIM1 in *E. coli* but found that it existed solely in inclusion bodies. We thus created two mutants, one equivalent to abLIM-s (namely herein ΔLIM) and the other containing only the VHP domain (Fig. 6a; also see Supplementary Fig. 1), and purified His-GFP, His-GFP-ΔLIM, and His-GFP-VHP from *E. coli* for in vitro assays (Fig. 6b).

When 3 μm of the purified proteins were added into preformed F-actin (Fig. 6c), we found that His-GFP-ΔLIM, but not His-GFP-VHP or His-GFP, distributed along actin filaments and potently induced intricate F-actin networks (Fig. 6d). Negative staining EM indicated that His-GFP-ΔLIM induced actin bundles and cross-linked them into networks (Fig. 6e). These results suggest that abLIM1 is a microfilament bundling and crosslinking protein capable of inducing F-actin network formation.

### abLIM1 associates with cell cortex proteins through its dematin-homologous region

**Fig. 7:**
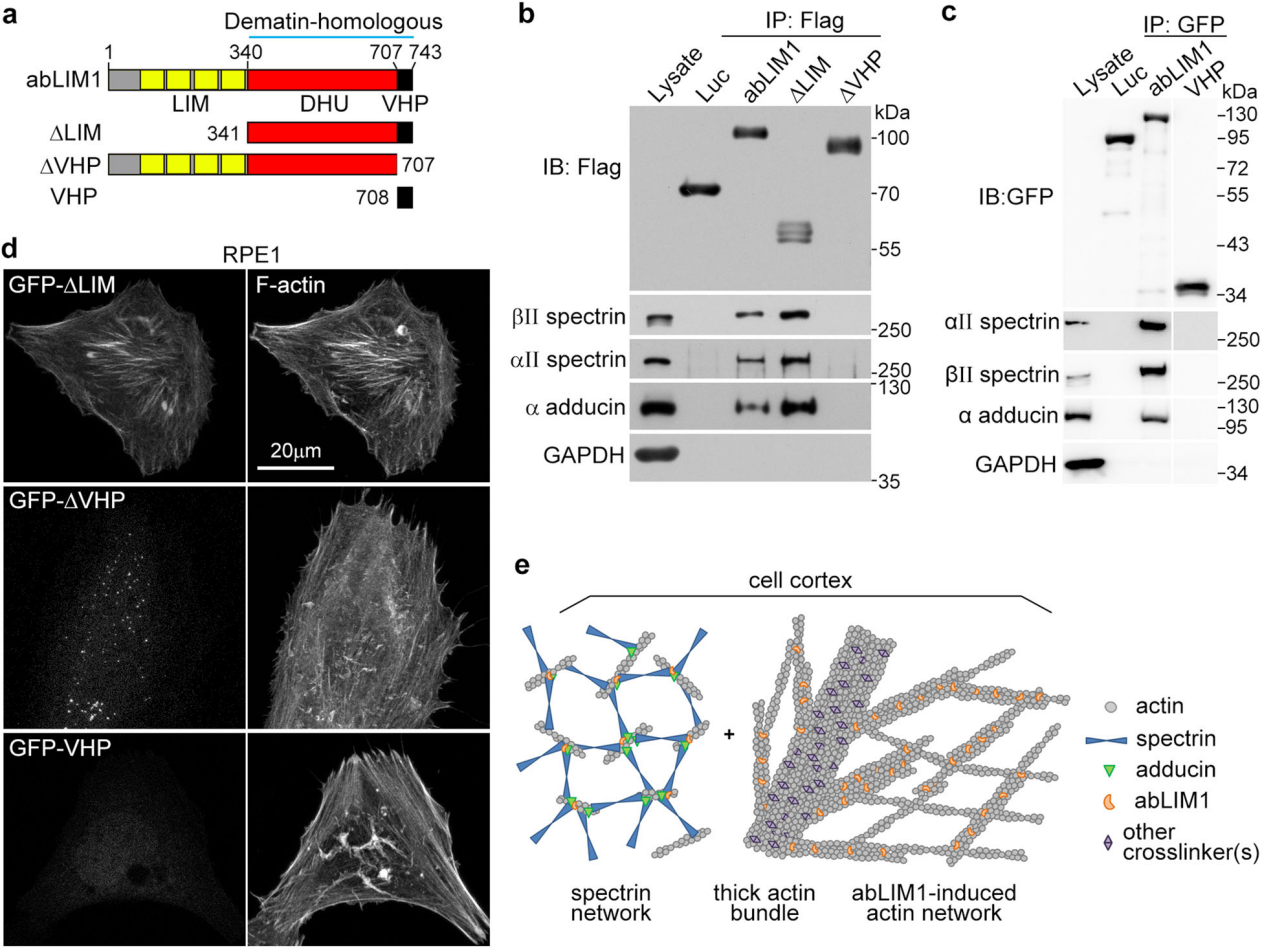
The dematin-homologous region of abLIM1 binds to both cortex proteins and cortical actin. **a** Diagrams of human abLIM1 and its mutants. LIM, LIM motif-containing region; DHU, dematin-homologous unfolded region; VHP, villin headpiece domain. **b, c** abLIM1 associated with cell cortex proteins spectrins and adducin through the dematin-homologous region. The indicated Flag-tagged (**b**) or GFP-tagged (**c**) proteins were expressed in HEK293T cells and subjected to co-IP. Exogenous luciferase (Luc) and endogenous GAPDH served as negative controls. All the lanes in (**c**) were from same blots. **d** Subcellular localization of GFP-tagged abLIM1 mutants. RPE1 cells were transfected for 24 h to express the indicated proteins and then stained for F-actin. **e** Model. In non-erythroid cells such as RPE1 and U2OS, the cortex is composed of a layer of spectrin network (only a few components are illustrated for simplicity) immediately underneath the plasma membrane, followed by dense intricate cortical actin networks organized by abLIM1 and other actin crosslinkers. For illustration purpose, the two types of cortical cytoskeleton networks are shown separately. Please see discussion for details

To understand how abLIM1 is targeted to the cortex, we performed co-immunoprecipitation (co-IP) by mixing Flag-abLIM1 or Flag-luciferase (a negative control) expressed in HEK293T cells with mouse brain lysates to identify its associated proteins, as done previously^41^. Silver staining indicated that many more proteins were associated with Flag-abLIM1 than with Flag-luciferase (Supplementary Fig. 6a). Subsequent shotgun mass spectrometry identified multiple cortex proteins such as αII and βII spectrins, α adducin, and ankyrin-2 (Supplementary Fig. 6a)^7^. To confirm the mass spectrometric results, we expressed Flag- or GFP-tagged abLIM1 or its mutants (Fig. 7a) in HEK293T cells and performed co-IP with the cell lysates. Immunoblotting using available antibodies confirmed the associations of endogenous spectrins and adducin with abLIM1 (Fig. 7b, c). These cortex proteins associated with Flag-ΔLIM but not Flag-ΔVHP or GFP-VHP (Fig. 7b, c), suggesting that the entire dematin-homologous region (equivalent to ΔLIM) is required for the associations. These cortex proteins readily displayed enhanced associations with Flag-ΔLIM but failed to associate with Flag-ΔVHP or GFP-VHP (Fig. 7b, c), suggesting that the entire dematin-homologous region (equivalent to ΔLIM) is required for the associations but the N-terminal LIM domain-containing region is inhibitory.

We then expressed these proteins as GFP fusions in RPE1 cells and examined their subcellular localizations. GFP-ΔLIM exhibited strong associations with bundled F-actin (Fig. 7d), similar to the full-length GFP-abLIM1 (Fig. 1d, e). By contrast, GFP-tagged ΔVHP and VHP displayed neither F-actin association nor spectrin-like enrichment at cell edges (Fig. 7d). Thus, the dematin-homologous region is also required for the association of abLIM1 with cortical actin filaments.

Finally, we investigated whether the depletion of abLIM1 affected the cortex localization of spectrin. We imaged spherical U2OS cells, induced by the EDTA treatment, with a confocal microscope (Supplementary Fig. 6b) and quantified relative immunofluorescent intensities using single optical sections at approximately the equator of the cells (Supplementary Fig. 6c). The average intensities of abLIM1 and βII spectrin were reduced by 81.5% and 30.2%, respectively, in the cells transfected with abL1-i1 as compared to the control cells (Supplementary Fig. 6c). Thus, abLIM1 also contributes to the cortical localization of spectrin.

## Discussion

We demonstrated that abLIM1 is a non-erythrocyte-specific cortex protein critical for the formation of dense interwoven cortical actin meshwork (Figs. 5 and 6). Such abLIM1-dependent actin networks antagonize cortical tensions generated by membrane protrusion, myosin-mediated contraction, and possibly other mechanical stresses (Figs. 2–4)^1, 3^. Accordingly, cells lacking abLIM1 displayed prominent blebbing during cell spreading or migration (Figs. 2–3). abLIM1 is widely expressed in cell lines and tissues (Fig. 1a)^17^ and may have similar functions in them. As overexpressed abLIM2 or abLIM3 also display colocalization with F-actin fibers^20, 23^, they may function similarly to abLIM1 in different cells or redundantly with abLIM1 in the same cells. Nevertheless, we found that abLIM1 is not required for the assembly and function of the cage-like cortical actin meshwork under the bleb membrane (Fig. 2f and Supplementary Video 2)^12, 42^.

abLIM1 may organize F-actin through similar mechanisms as dematin. An F-actin crosslinker must have two actin binding sites, achieved through either two intrinsic sites or dimerization when there is only one^7, 43, 44^. Dematin is known to contain two actin-binding sites, one in its N-terminal unfolded region and the other in the VHP domain^45, 46^. We found that the entire dematin-homologous region of abLIM1, i.e., ΔLIM, also binds to and crosslinks F-actin into networks (Figs. 6 and 7d), suggesting that it contains two actin-binding sites as well. Nevertheless, unlike dematin, the VHP domain of abLIM1 alone does not show detectable binding to F-actin both in vitro and in vivo (Figs. 6 and 7d). The failure of ΔVHP, a mutant lacking only the VHP domain, to associate with F-actin in cells (Fig. 7a, d) suggests that the dematin-homologous unfolded region of abLIM1 alone (termed herein as the DHU region; Fig. 7a) is not sufficient for binding to F-actin, either. As VHP domains from different proteins can show diverse actin-binding activities in vitro^47^, our results imply that the VHP and DHU regions of abLIM1 may need to stay together to augment each other’s actin binding activities so that ΔLIM can bind and crosslink F-actin. In addition, abLIM1 may be regulated by mechanisms distinct from dematin. For instance, the F-actin crosslinking activity of dematin is negatively regulated by protein kinase A-mediated phosphorylation of Ser381 in the VHP domain^46, 48, 49^. This site, however, is not conserved in abLIM proteins. On the other hand, DYRK1A kinase is reported to negatively regulate abLIM1^24, 25^.

Our results suggest that abLIM1 is recruited to the cortical spectrin network through association with spectrins and/or spectrin-associated proteins such as adducin (Fig. 7 and Supplementary Fig. 6a). In red blood cells the cortex is mainly formed by αI and βI spectrin tetramers, crosslinked by short actin filaments and associated cortical actin-binding proteins such as adducin and dematin, and anchored to the plasma membrane through other cortex components such as ankyrin^5, 7, 9^. Dematin is located at the junction of the spectrin tetramer and F-actin and can facilitate the spectrin-actin interactions^5, 49^. *Dematin* deficiency in mice reduced erythroid membrane-associated spectrin and adducin and caused extensive bleb formation in erythrocytes and severe anemia^50^. Similarly, in non-erythroid cells we found that depletion of abLIM1 by RNAi led to bleb formation and reduced cortical βII spectrin (Figs. 2, 3 and Supplementary Fig. 6b, c). Therefore, abLIM1 might be similarly recruited to and function in the spectrin network (Fig. 7e). Furthermore, it can also assemble an additional set of dense cortical F-actin networks peculiar to the non-erythroid cells (Figs. 5a, d and 7e)^10, 13^.

The dense intricate cortical actin networks are organized by abLIM1 and probably other actin crosslinker(s)^43^ as well (Fig. 7e). abLIM1 was not detected on the thick cortical actin bundles at endogenous or low exogenous levels unless being highly expressed (Fig. 1 and Supplementary Fig. 2e). Furthermore, depletion of abLIM1 impaired the hierarchically branching actin networks but augmented the thick actin bundles (Fig. 5). These observations suggest that such thick bundles are assembled by unidentified actin crosslinker(s) and integrated into the abLIM1-induced actin network (Fig. 7e). This also explains why the overall cortical actin thickness was not reduced after the depletion of abLIM1 (Supplementary Fig. 5). mDia1 and the Arp2/3 complex have been shown to control the nucleation of approximately 50% of the cage-like cortical actin meshwork formed under the bleb membrane and are also important for the “conventional” cortical actin^42, 51^. As they respectively nucleate linear and branched actin filaments^52^, abLIM1 and other actin crosslinker(s) may further organize these actin filaments into complicated cortical actin networks (Figs. 5a, d and 7e)^13^. Therefore, the relative levels or activities of these cortical actin regulators may determine both the overall and regional structural characteristics of the cell cortex and consequently modulate its functions in accordance to different cellular activities.

Interestingly, there are multiple isoforms of human abLIM1. Roof et al. report three isoforms identified from a human retina cDNA library, the full-length protein of 778 amino acid residues termed abLIM-I, an intermediate form containing three LIM motifs (abLIM-m), and a short one lacking any LIM domain (abLIM-s)^17^. Their PCR assays suggest that abLIM-I is only expressed in a portion of tissues such as retina, whereas abLIM-s is widely expressed^17^. The full-length cDNA that we cloned from RPE1 cells encoded 743 amino acids (Supplementary Fig. 1), lacking 35 amino acid residues in the dematin-homologous region. However, our exogenous full-length abLIM1 with either Flag-, Flag-HA- or Myc-tag migrated above the 100-kDa marker in SDS-PAGE, whereas the major band of endogenous abLIM1 in RPE1 or U2OS cells migrated between the 70-kDa and 100-kDa markers (see Figs. 1a, 2g, h and 7b for comparisons)^23^. As Flag-ΔLIM migrated between the 55 and 70 kDa markers (Fig. 7b), we suspect that the major isoform expressed in RPE1 and U2OS cells is the one similar to abLIM-m. This isoform is also the major one expressed in IMCD3 and NIH3T3 cells and the brain tissue, whereas HEK293T and HeLa cells appeared to also express the full-length abLIM1 (Fig. 1a). Our co-IP results suggest that the LIM-containing region of abLIM1 is inhibitory to the associations with spectrins and adducin (Fig. 7b). Its detailed roles, as well as the functional difference of the aforementioned isoforms thus remain to be clarified.

## Materials and methods

### Plasmids and oligonucleotides

The full-length abLIM1 and abLIM3 cDNAs (GenBank accession number MF597763, NM_001345859) were cloned from RPE1 cells^23^. Deletion mutants of abLIM1 were generated by PCR. The RNAi-resistant abLIM1 (abLIM1R) cDNA was created by making point mutations that did not affect the coding amino acids in the abL1-i1 target site. Vectors pEGFP-C, pFlag-CMV-2, and pET28a were used to express GFP-tagged, Flag-tagged, and His-tagged proteins, respectively. The lentiviral constructs of GFP-F and RFP-Utrch^30, 32^ were constructed by replacing the GFP cassette of pLV-GFP-C1 with the coding sequence of GFP-F and RFP-Utrch, respectively. Lentiviral vector pFUGW (which contains IRES-EGFP as an expression marker) was used to express Flag-HA-tagged abLIM1R and luciferase for rescue experiments. All the constructs were verified by sequencing. The siRNAs were ordered from GenePharma and their sequences were described previously^23^.

### Antibodies

Primary antibodies against the following proteins or peptides were used: α-tubulin (Sigma T5168, mouse, 1:5000), β-actin (Sigma A5316, mouse, 1:5,000), Flag-M2 (Sigma F3165, mouse, 1:5,000; Sigma F7425, rabbit, 1:5,000), βII spectrin (BD 612563, mouse, 1:10,000 for immunoblotting and 1:40,000 for immunostaining), αII spectrin (BD 612560, mouse, 1:2,000), α adducin (Santa Cruz sc-25731, rabbit, 1:2,000), GAPDH (Proteintech 10494-1-AP, rabbit, 1:10,000), GFP (Santa Cruz sc-8334, rabbit, 1:3,000), ERMs (CST 3142, rabbit, 1:1,000), phospho-ERMs (CST 3726, rabbit, 1:1,000), MLC2 (CST 3672, Rabbit, 1:500), phospho-MLC2(Ser19) (CST 3675, mouse, 1:1,000), phospho-MLC2(Thr18/Ser19) (CST 3674, rabbit, 1:1,000), and abLIM1 (home-made, rabbit, 1:5,000 for immunoblotting and 1:500-2,000 for immunostaining). Secondary antibodies conjugated with Alexa Fluor-488, -546, -647 (1:1,000) or horse radish peroxidase (1:5,000) were purchased from Invitrogen.

### Cell culture, transfection, and virus infection

hTERT-RPE1 cells were maintained in Dulbecco’s Modified Eagle Medium:Nutrient Mixture F-12 (DMEM/F12) media (ThermoFisher) supplemented with 10% fetal bovine serum (FBS), 100 units/ml penicillin, 100 μg/ml streptomycin, and 10 μg/ml Hygromycin B (Invitrogen). HEK293T and U2OS cells were maintained in DMEM supplemented with the same amount of FBS, penicillin, and streptomycin. For plasmid transfection, the conventional calcium phosphate method was used for HEK293T cells and Lipofectamine 2000 (Invitrogen) for RPE1 and U2OS cells. Lipofectamine RNAiMAX (Invitrogen) was used for siRNA transfection. Lentiviral particles were packaged as described previously^53^. The virus-infected RPE1 cells were isolated by FACS based on expressed fluorescent marker(s) and cultured as stable cells. In this study, HEK293T cells were mainly used for biochemical assays due to their high transfection efficiency for plasmids.

### Light microscopy

For immunofluorescent microscopy, RPE1 or U2OS cells were cultured on coverslips and fixed with 4% paraformaldehyde in PBS for 15 min at room temperature. To induce a spherical morphology, the cells were partially detached from the substratum by treating with 0.5 mM EDTA in PBS for 10 min at 37 °C prior to the fixation. For immunostaining concerning abLIM1, the cells were pre-extracted with 0.1% Triton X-100 in PBS for 30–60 sec prior to the fixation. The fixed cells were permeabilized with 0.5% Triton X-100 in PBS for 10 min and blocked with 4% BSA in TBST for 1 h. The incubation with primary and secondary antibodies was carried out at 4 °C overnight and at room temperature for 1 h. Phalloidin-TRITC (Sigma, P1951, 1:1,000) or phalloidin-Alexa Fluor-647 (Invitrogen, A22287, 1:1,000) was used to stain F-actin. Nuclear DNA was stained with 4, 6-diamidino-2-phenylindole (DAPI). Optical sections were captured at 0.3-μm intervals using a confocal microscope (TCS SP8, Leica). The resulting z-stack images were rendered 2D by maximum intensity projections unless otherwise stated.

For time-lapse microscopy, RPE1 cells were maintained in L-15 medium (Invitrogen) supplemented with 10% fetal bovine serum, 100 units/ml penicillin, and 100 μg/ml streptomycin. Image sequences for cell migration or spreading were collected at 6-min intervals for 10 h using a CCD camera (Evolution QEi, Media Cybernetics) on an Olympus IX81 microscope equipped with a motorized stage and a 37 °C heating chamber. Blebbing dynamics was traced with a spinning disc microscope (UltraVIEW VoX, PerkinElmer) at 10-sec intervals for 30 min.

STED images of abLIM1 (Alexa Fluor-546) and F-actin (Alexa Fluor-647) were acquired on a Leica TCS SP8 STED ×3 microscope with 660 nm and pulsed 775 nm lasers for depletion, 546 nm and 647 nm of a pulsed white light laser (WLL) for excitation, respectively. The gating time for both STED channels ranged from 0.5 ns to 6 ns. A Leica HC PL APO CS2 ×100/1.40 oil objective was used.

### Scanning electron microscopy

For imaging cell surface, RPE1 cells grown on glass coverslips were fixed for 1.5 h or overnight at 4 °C with 2.5% glutaraldehyde in PBS, followed by three rounds of wash (20 min each) with PBS and then a post-fixation in 1% osmic acid for 1 h. After three times of wash (10 min each), the samples were dehydrated by exposure to serial ethanol dilutions and dried in a critical point dryer. They were coated with 10-nm to15-nm platinum and imaged with a scanning EM (FEI Quanta 250).

For imaging cortical actin, RPE1 cells were processed as described^38^ with minor modifications. Briefly, the cells cultured on glass coverslips were washed quickly with PBS pre-warmed to 37 °C and transferred to PEM buffer (100 mM PIPES (pH 6.9), 1 mM EGTA, and 1 mM MgCl_2_) containing 1% Triton X-100 for 5 min. After two rounds of wash in PEM buffer, the cells were fixed in PEM buffer containing 2% glutaraldehyde for 20 min. The cells were then dehydrated by exposure to serial ethanol dilutions, dried in a critical point dryer, and coated with 5 to 7-nm platinum prior to imaging.

### Atomic force microscopy

Imaging and cell elasticity measurements were performed using a Bioscope Resolve atomic force microscope (Bruker, Santa Barbra, CA), operating in the PeakForce live cell mode in DMEM at room temperature. Olympus AC40-TS cantilevers (tip length 40 μm, tip radius 10 nm, nominal spring constant 0.09 N/m) were used in all experiments. Before each measurement, we performed a force-versus-distance curve on the petri dish adjacent to the cells to measure the detection sensitivity and determined the spring constant of the cantilever by recording the thermal noise power spectrum in liquid. For all measurements, the scan rate was 0.3 Hz and 0.4 Hz for whole cell and high resolution imaging of the cortex, respectively, with a frame of 128×128 pixels, using a PeakForce frequency of 1 kHz and an oscillation amplitude of 100 nm. The force setpoint was set to 150 pN and an automatic gain control was used to minimize the PeakForce error. Image analysis was performed using the Nanoscope analysis v1.80 (Bruker Nano Surfaces, Santa Barbara).

### Protein expression and purification

Expressions of His-GFP-tagged proteins in *E. coli* were induced with 0.2 mM IPTG at 16°C for 20 h. Protein purification was performed at 4 °C according to manufacturer’s protocol (Qiagen, Sigma). Briefly, *E. coli* was lysed in the lysis buffer [20 mM Tris-HCl (pH 8.0), 300 mM NaCl, 5% glycerol, 5 mM imidazole, 1% TritonX-100, 1 mM phenylmethylsulfonyl fluoride (PMSF), and protease inhibitor cocktail (Calbiochem)] using high pressure homogenizer (JNBIO JN-02C). After centrifugation to remove debris, the lysates were incubated with Ni-NTA beads (Qiagen) for 2 h. Then the beads were washed with wash buffer (20 mM Tris-HCl (pH 8.0), 300 mM NaCl, 5% glycerol, 20–70 mM imidazole) for 50–75 × beads volumes and eluted with elution buffer (20 mM Tris-HCl (pH 8.0), 300 mM NaCl, 5% glycerol, 300 mM imidazole). The eluted proteins were dialyzed twice with large volumes of PBS for 1 h each, followed by concentration using spin columns (Amicon 10-kDa ultra centrifugal filters, Millipore) following manufacturer’s protocol. For proteins susceptible to precipitation during the dialysis, we slowed down the process by capping them in Eppendorf tubes with 14-kDa dialysis membrane, followed by dialyzing in PBS twice for approximately 10 h each. His-abLIM1 and -abLIM3 were purified as inclusion bodies, solubilized in 8 M urea, and dialyzed. The purified proteins were divided into 5- or 10-μl aliquots, snap frozen in liquid nitrogen, and stored at −80 °C.

### F-actin bundling assays

Non-muscle actin (1 mg/ml; Cytoskeleton Inc.) in 5 mM Tris-HCl (pH 7.5), 0.2 mM CaCl_2_, and 0.2 mM ATP was mixed with 0.1 volume of 10× polymerization buffer [100 mM Tris-HCl (pH 7.5), 500 mM KCl, 20 mM MgCl_2_, and 10 mM ATP] and incubated at 25 °C for 1 h to polymerize F-actin. 6 μl of the F-actin-containing solution (containing 22 μm actin) were then mixed with 16 μl of different concentrations of His-GFP-tagged proteins and incubated at 25 °C for 1 h. An aliquot of each mixture was stained with phalloidin-TRITC (0.001 μg/μl final concentration; Sigma-Aldrich) for 15 min at 25 °C and mounted into a 35-mm Petri dish with cover glass bottom for confocal microscopy. The remaining aliquot of the mixture was loaded onto carbon-coated and glow-discharged copper grids (SPI Supplies) for 1 min, washed once with 0.75% uranyl formate, and stained with 0.75% uranyl formate for 60 s. Then the sample was air-dried and imaged in a transmission electron microscope (FEI Tecnai G2 Spirit) operated at 120 kV.

### Co-immunoprecipitation

Cells cultured in a 10-cm Petri dish were lysed on ice in 1 ml of lysis buffer [20 mM Tris-Cl (pH 7.5), 100 mM KCl, 0.1% NP-40, 1 mM EDTA, 10% glycerol, 10 mM Na_4_P_2_O_7_, 1 mM Na_3_VO_4_, 50 mM NaF, 1 mM phenylmethylsulfonyl fluoride, 1 mM DTT, and protease inhibitor cocktail (Calbiochem)]. The lysates were pre-cleared by centrifugation at 14,000×*g* for 10 min. The supernatants were mixed with 20 μl 50% slurry of anti-Flag M2 resin (Sigma) or anti-GFP resin (Chromotek) and incubated at 4 °C for 2 h in a rotary station. After three times of wash with the lysis buffer and then with wash buffer [20 mM Tris-Cl (pH 7.5), 150 mM KCl, 0.5% NP-40, 1 mM EDTA, 10% glycerol, 10 mM Na_4_P_2_O_7_, 1 mM Na_3_VO_4_, 50 mM NaF, 1 mM phenylmethylsulfonyl fluoride, and 1 mM DTT], proteins on the anti-Flag beads were eluted using 30 μl of 1 mg/ml Flag peptide, whereas those on the anti-GFP beads were solubilized using SDS loading buffer. To prepare samples for shotgun mass spectrometry, lysates from HEK293T cells transfected for 48 h to express Flag-tagged abLIM1 or luciferase were mixed with P0 mouse brain lysates and subjected to co-IP as described previously^41^.

### Quantification and statistical analysis

Tracks of the nucleus over 600 min, generated using ImageJ software (NIH), were used as trajectories of migrating cells. The length of the straight line between the initial (0 min) and end (600 min) positions of a trajectory was measured using ImageJ as the net displacement of the cell.

To determine the Young’s modulus of the cells with AFM, we employed the Dejarguin, Muller, Toporov (DMT) model^54^ using the equation,

$F = \frac{{4E}}{{3\left( {1 - v^2} \right)}}\sqrt R d^{\frac{3}{2}} + F_{\mathrm{adh}}$where *F* is the force applied, *F*_adh_ is the adhesion force measured from the force curve, *E* is the Young’s modulus, *R* is the tip radius, *d* is the indentation depth, and *v* is the Poisson ratio (0.5)^55, 56^. *F*_adh_ was measured during PeakForce imaging. For all measurements, we examined individual approximately 10×10-µm^2^ regions isolated from the whole cell data. For the regions away from the nucleus, we limited our examinations to those sufficiently far from the cell boundary to avoid contributions of the underlying substrate to the elasticity measurements in the thinner regions of the cell.

To measure cell cortex thickness, STED images of single optical section at approximately the equatorial position of U2OS cells were used. Three separate fluorescence intensity line scans were performed across the border of a cell at where the cortex staining was relatively uniform (see Supplementary Fig. 5b). Average width of the intensity curves at the 50% position of the intensity peak was used as the cortex thickness of the cell.

Unpaired Student’s *t* -test was performed for statistical analysis by using SigmaPlot (Systat Software, Inc.). Differences were considered significant when *P* < 0.05.

### Data availability

Data supporting the reported results are available upon request to X.Z.

## Electronic supplementary material

Blebbing of abLIM1-depleted RPE1 cells during cell spreading

Detailed blebbing process of abLIM1-depleted spreading cells

Blebbing of abLIM1-depleted RPE1 cells during cell migration

Supplementary information

Readme file for supplementary information

## References

[1] Salbreux G, Charras G, Paluch E (2012). Actin cortex mechanics and cellular morphogenesis. Trends Cell Biol..

[2] Andrade DM (2015). Cortical actin networks induce spatio-temporal confinement of phospholipids in the plasma membrane--a minimally invasive investigation by STED-FCS. Sci. Rep..

[3] Koster DV, Mayor S (2016). Cortical actin and the plasma membrane: inextricably intertwined. Curr. Opin. Cell Biol..

[4] Charras G, Paluch E (2008). Blebs lead the way: how to migrate without lamellipodia. Nat. Rev. Mol. Cell Biol..

[5] Machnicka B, Grochowalska R, Boguslawska DM, Sikorski AF, Lecomte MC (2012). Spectrin-based skeleton as an actor in cell signaling. Cell Mol. Life Sci..

[6] Wu SK (2014). Cortical F-actin stabilization generates apical-lateral patterns of junctional contractility that integrate cells into epithelia. Nat. Cell Biol..

[7] Baines AJ (2010). The spectrin-ankyrin-4.1-adducin membrane skeleton: adapting eukaryotic cells to the demands of animal life. Protoplasma.

[8] Fritzsche M (2017). Cytoskeletal actin dynamics shape a ramifying actin network underpinning immunological synapse formation. Sci. Adv..

[9] Bennett V, Baines AJ (2001). Spectrin and ankyrin-based pathways: metazoan inventions for integrating cells into tissues. Physiol. Rev..

[10] Fritzsche M, Lewalle A, Duke T, Kruse K, Charras G (2013). Analysis of turnover dynamics of the submembranous actin cortex. Mol. Biol. Cell.

[11] Ridley AJ (2011). Life at the leading edge. Cell.

[12] Charras GT, Hu CK, Coughlin M, Mitchison TJ (2006). Reassembly of contractile actin cortex in cell blebs. J. Cell Biol..

[13] Li D (2015). ADVANCED IMAGING. Extended-resolution structured illumination imaging of endocytic and cytoskeletal dynamics. Science.

[14] Xu K, Zhong G, Zhuang X (2013). Actin, spectrin, and associated proteins form a periodic cytoskeletal structure in axons. Science.

[15] D’Este E, Kamin D, Gottfert F, El-Hady A, Hell SW (2015). STED nanoscopy reveals the ubiquity of subcortical cytoskeleton periodicity in living neurons. Cell Rep..

[16] Sidenstein SC (2016). Multicolour multilevel STED nanoscopy of actin/spectrin organization at synapses. Sci. Rep..

[17] Roof DJ, Hayes A, Adamian M, Chishti AH, Li T (1997). Molecular characterization of abLIM, a novel actin-binding and double zinc finger protein. J. Cell Biol..

[18] Rana AP, Ruff P, Maalouf GJ, Speicher DW, Chishti AH (1993). Cloning of human erythroid dematin reveals another member of the villin family. Proc. Natl Acad. Sci. USA.

[19] Azim AC, Knoll JH, Beggs AH, Chishti AH (1995). Isoform cloning, actin binding, and chromosomal localization of human erythroid dematin, a member of the villin superfamily. J. Biol. Chem..

[20] Barrientos T (2007). Two novel members of the ABLIM protein family, ABLIM-2 and -3, associate with STARS and directly bind F-actin. J. Biol. Chem..

[21] Krupp M, Weinmann A, Galle PR, Teufel A (2006). Actin binding LIM protein 3 (abLIM3). Int J. Mol. Med.

[22] Frank D, Frey N (2011). Cardiac Z-disc signaling network. J. Biol. Chem..

[23] Cao J (2012). miR-129-3p controls cilia assembly by regulating CP110 and actin dynamics. Nat. Cell Biol..

[24] Schneider P (2015). Identification of a novel actin-dependent signal transducing module allows for the targeted degradation of GLI1. Nat. Commun..

[25] Schneider P (2015). Corrigendum: identification of a novel actin-dependent signal transducing module allows for the targeted degradation of GLI1. Nat. Commun..

[26] Shan Y (2009). Nudel and FAK as antagonizing strength modulators of nascent adhesions through paxillin. PLoS Biol..

[27] Mitra SK, Hanson DA, Schlaepfer DD (2005). Focal adhesion kinase: in command and control of cell motility. Nat. Rev. Mol. Cell Biol..

[28] Charras GT (2008). A short history of blebbing. J. Microsc..

[29] Tinevez JY (2009). Role of cortical tension in bleb growth. Proc. Natl Acad. Sci. USA.

[30] Jiang W, Hunter T (1998). Analysis of cell-cycle profiles in transfected cells using a membrane-targeted GFP. Biotechniques.

[31] Liang Y (2004). Nudel functions in membrane traffic mainly through association with Lis1 and cytoplasmic dynein. J. Cell Biol..

[32] Burkel BM, von Dassow G, Bement WM (2007). Versatile fluorescent probes for actin filaments based on the actin-binding domain of utrophin. Cell Motil. Cytoskel..

[33] Bergert M, Chandradoss SD, Desai RA, Paluch E (2012). Cell mechanics control rapid transitions between blebs and lamellipodia during migration. Proc. Natl Acad. Sci. USA.

[34] Straight AF (2003). Dissecting temporal and spatial control of cytokinesis with a myosin II Inhibitor. Science.

[35] Watanabe T, Hosoya H, Yonemura S (2007). Regulation of myosin II dynamics by phosphorylation and dephosphorylation of its light chain in epithelial cells. Mol. Biol. Cell.

[36] Sakurada K, Seto M, Sasaki Y (1998). Dynamics of myosin light chain phosphorylation at Ser19 and Thr18/Ser19 in smooth muscle cells in culture. Am. J. Physiol..

[37] Fehon RG, McClatchey AI, Bretscher A (2010). Organizing the cell cortex: the role of ERM proteins. Nat. Rev. Mol. Cell Biol..

[38] Svitkina T (2007). Electron microscopic analysis of the leading edge in migrating cells. Methods Cell Biol..

[39] Zhang Y (2017). In vivo dynamics of the cortical actin network revealed by fast-scanning atomic force microscopy. Microscopy.

[40] Vicidomini G (2011). Sharper low-power STED nanoscopy by time gating. Nat. Meth.

[41] Shen Y (2008). Nudel binds Cdc42GAP to modulate Cdc42 activity at the leading edge of migrating cells. Dev. Cell.

[42] Bovellan M (2014). Cellular control of cortical actin nucleation. Curr. Biol..

[43] Revenu C, Athman R, Robine S, Louvard D (2004). The co-workers of actin filaments: from cell structures to signals. Nat. Rev. Mol. Cell Biol..

[44] Chhabra ES, Higgs HN (2007). The many faces of actin: matching assembly factors with cellular structures. Nat. Cell Biol..

[45] Chen L, Jiang ZG, Khan AA, Chishti AH, McKnight CJ (2009). Dematin exhibits a natively unfolded core domain and an independently folded headpiece domain. Protein Sci..

[46] Chen L, Brown JW, Mok YF, Hatters DM, McKnight CJ (2013). The allosteric mechanism induced by protein kinase A (PKA) phosphorylation of dematin (band 4.9). J. Biol. Chem..

[47] Vardar D (2002). Villin-type headpiece domains show a wide range of F-actin-binding affinities. Cell Motil. Cytoskel..

[48] Husain-Chishti A, Levin A, Branton D (1988). Abolition of actin-bundling by phosphorylation of human erythrocyte protein 4.9. Nature.

[49] Koshino I, Mohandas N, Takakuwa Y (2012). Identification of a novel role for dematin in regulating red cell membrane function by modulating spectrin-actin interaction. J. Biol. Chem..

[50] Lu Y (2016). Gene disruption of dematin causes precipitous loss of erythrocyte membrane stability and severe hemolytic anemia. Blood.

[51] Fritzsche M, Erlenkamper C, Moeendarbary E, Charras G, Kruse K (2016). Actin kinetics shapes cortical network structure and mechanics. Sci. Adv..

[52] Campellone KG, Welch MD (2010). A nucleator arms race: cellular control of actin assembly. Nat. Rev. Mol. Cell Biol..

[53] Zhao H (2013). The Cep63 paralogue Deup1 enables massive de novo centriole biogenesis for vertebrate multiciliogenesis. Nat. Cell Biol..

[54] Boer-Duchemin E, Tranvouez E, Dujardin G (2010). The interaction of an atomic force microscope tip with a nano-object: a model for determining the lateral force. Nanotechnology.

[55] Eghiaian F, Rigato A, Scheuring S (2015). Structural, mechanical, and dynamical variability of the actin cortex in living cells. Biophys. J..

[56] Trickey WR, Baaijens FP, Laursen TA, Alexopoulos LG, Guilak F (2006). Determination of the Poisson’s ratio of the cell: recovery properties of chondrocytes after release from complete micropipette aspiration. J. Biomech..

