# Supplementary material for: abLIM1 constructs non-erythroid cortical actin networks to prevent mechanical tension-induced blebbing
Source: Cell Discov. 2018 Jul 24;4:42. doi: 10.1038/s41421-018-0040-3 (PMC6056535; doi:10.1038/s41421-018-0040-3)
Supplement: Supplementary file 4 — Supplementary information [file 41421_2018_40_MOESM4_ESM.pdf]

Supplementary information

Supplementary Figures

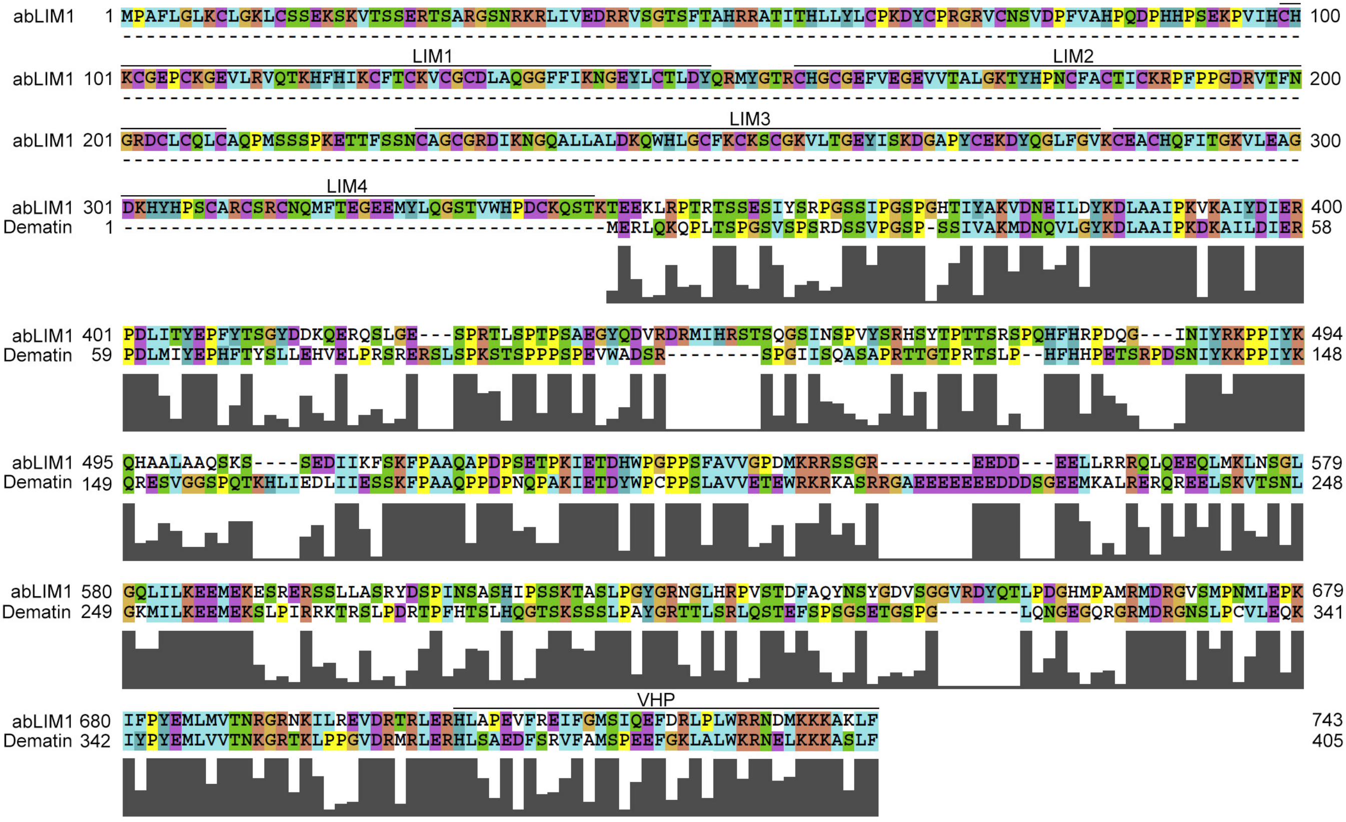

Supplementary Figure 1 Sequence alignment of human abLIM1 (GenBank accession MF597763) and dematin (NP_001107608) using ClustalW 2.0. Histograms indicate the extent of conservation. Positions of the LIM motifs (LIM1-4) and VHP domain are marked.

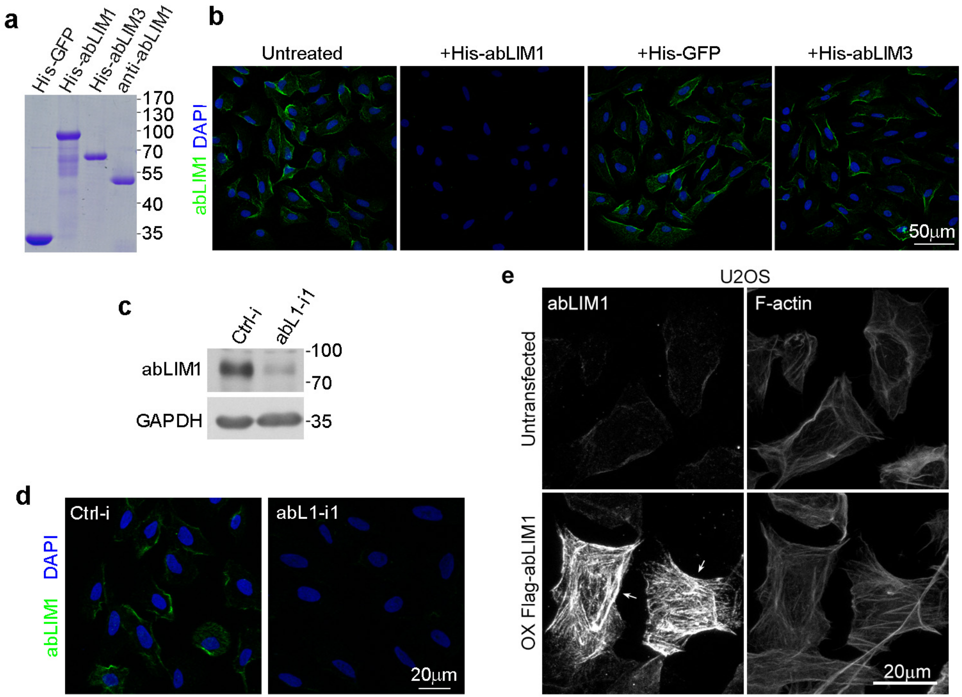

Supplementary Figure 2 Specificity of anti-abLIM1 antibody. **(a)** His-tagged proteins used for antibody neutralization experiments. His-tagged GFP, abLIM1, or abLIM3 purified from *E. coli* were subjected to SDS-PAGE with affinity-purified rabbit anti-abLIM1 antibody. The gel was stained with Coomassie Blue. **(b)** His-abLIM1, but not His-GFP or His-abLIM3, neutralized the antibody in immunostaining. The anti-abLIM1 antibody was either untreated or pre-incubated with PVDF membranes containing the indicated His-tagged proteins at a 1:500 molar ratio for 4 hr at room temperature. Immunostaining was then performed with U2OS cells. **(c,d)** RNAi markedly diminished the abLIM1 signals in both immunoblotting and immunostaining. U2OS cells were transfected with a control siRNA (Ctrl-i) or an abLIM1-specific siRNA (abL1-i1) for 72 hr. Immunoblotting (**c**) or immunostaining (**d**) was then performed to detect abLIM1. GAPDH served as loading control. DNA was stained with DAPI. **(e)** The antibody recognized exogenous abLIM1. U2OS cells that were either untransfected or transiently transfected to overexpress (OX) Flag-abLIM1 (arrows) were immunostained with anti-abLIM1 antibody. F-actin was labeled with phalloidin-TRITC. Note that abLIM1 showed different distribution patterns when overexpressed.

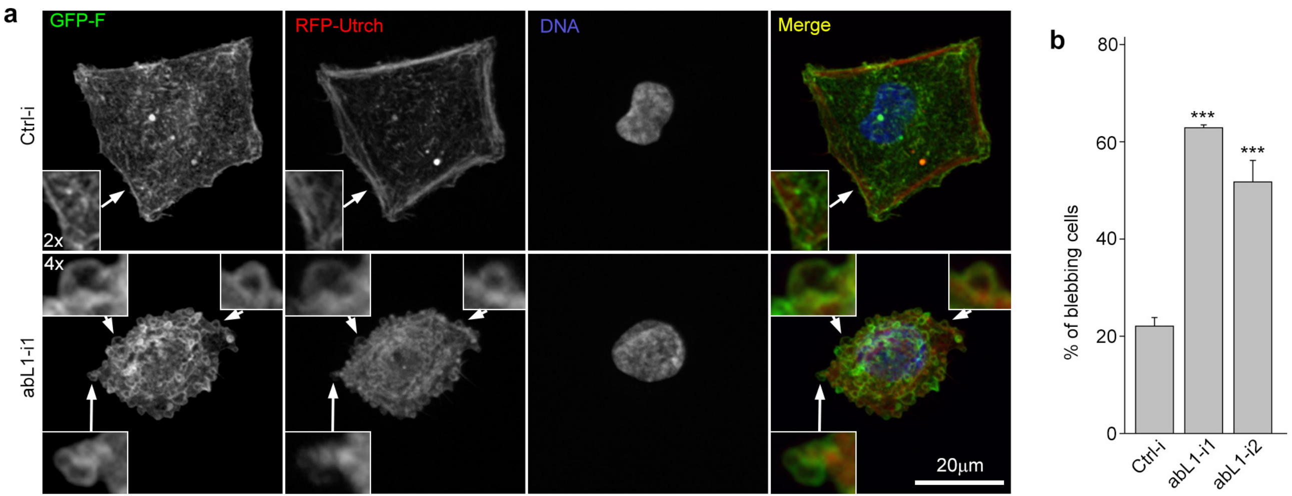

Supplementary Figure 3 GFP-F and RFP-Utrch respectively label the plasma membrane and F-actin. **(a)** RPE1 cells stably coexpressing GFP-F and RFP-Utrch were transfected for 48 hr with siRNA and re-plated at 10% confluency. The cells were fixed at 4 hr after the re-plating and stained with DAPI, followed by confocal microscopy. Magnified views are provided to show details. **(b)** Quantifications on blebbing cells fixed as in (**a**). The results were from three independent experiments. At least 50 cells were scored in each experiment and condition. Data are presented as mean ± s.d. Student's *t* test: *** $P<0.001$.

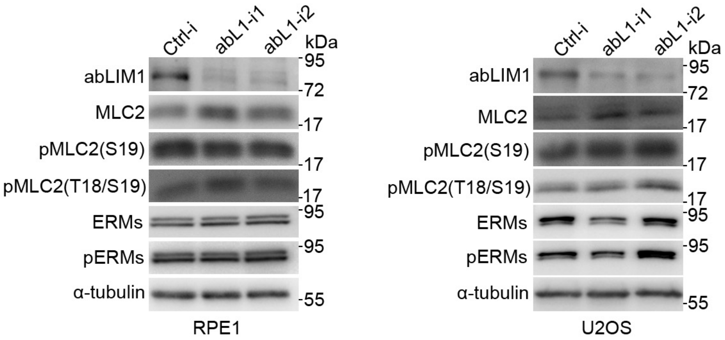

Supplementary Figure 4 Depletion of abLIM1 did not affect gross activities of myosin II and ERMs. RPE1 and U2OS cells transfected with siRNA (Ctrl-i, abL1-i1, or abL1-i2) for 48 hr were used for immunoblotting. α-tubulin served as loading control. pMLC2(S19), phosphorylated myosin light chain 2 at Ser19; pMLC2(T18/S19), phosphorylated myosin light chain 2 at Thr18 and Ser19; ERMs, Ezrin\Radixin\Moesin proteins; pERMs, phosphorylated Ezrin (Thr567)/Radixin (Thr564)/Moesin (Thr558).

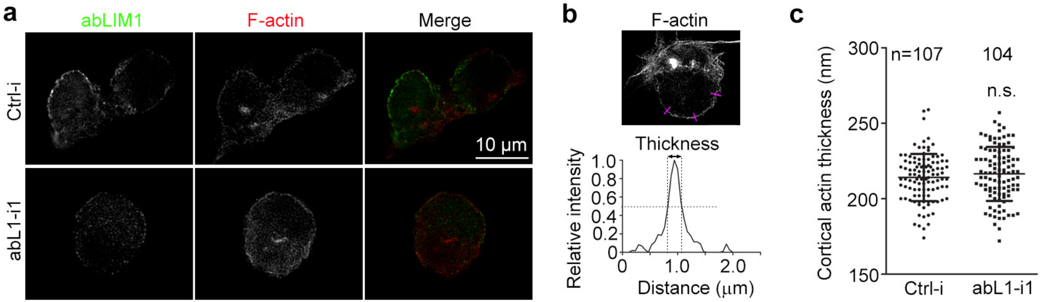

Supplementary Figure 5 abLIM1 depletion did not affect cortical actin thickness. **(a)** Representative STED images of single optical section at the equatorial position. U2OS cells transfected with Ctrl-i or abL1-i1 for 48 hr were treated with EDTA to allow roundup prior to fixation and fluorescent labeling. **(b)** Measurement of cortex actin thickness. Purple lines indicate the positions of line scans in the example cell. A thickness was measured from the fluorescence intensity curves at each position as shown. The average thickness of the three positions was used as cortex actin thickness of the cell. **(c)** Quantification results from cells imaged in three independent experiments. At least 33 cells were measured in each experiment and condition. The mean values and errors (s.d.) are also shown. n.s., no significance in Student's *t* test.

a

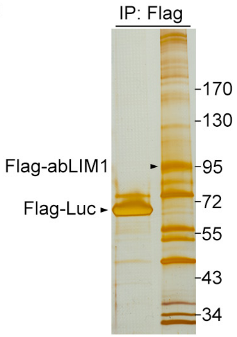

Protein name	Peptide count	Unique peptide count	Coverage (%)	MW (kDa)
Ankyrin-2	4	2	0.9%	433
αII spectrin	12	9	5.1%	285
βII spectrin	10	6	3.9%	274
α actinin-4	2	2	3.1%	105
α adducin	3	2	3.0%	81
Tropomyosin-3	8	3	13.7%	33
Tropomyosin-4	1	1	6.9%	28
Tropomyosin-1	5	3	13.1%	28

b

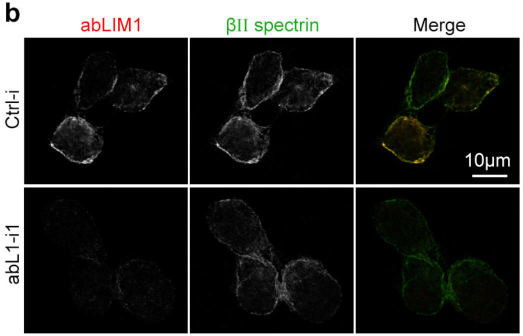

c

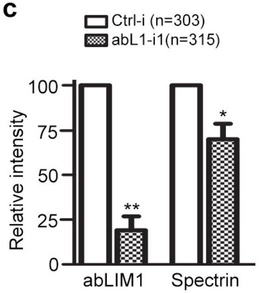

Supplementary Figure 6 Identification of abLIM1-associated cortex proteins. **(a)** P0 mouse brain lysates were mixed with Flag-tagged abLIM1 or Luciferase expressed in HEK293T cells and subjected to co-IP using anti-Flag resin. A portion of the eluted immunoprecipitates was separated by SDS-PAGE and visualized by silver staining. The remaining was analyzed by shotgun mass spectrometry. Cell cortex-related proteins appeared solely in the Flag-abLIM1 sample are listed in the table. **(b,c)** Depletion of abLIM1 moderately attenuated cortical spectrin. U2OS cells were transfected with ctrl-i or abL1-i1 for 48 hr and treated with 0.5 mM EDTA in PBS for 10 min to become round-up prior to fixation. Representative confocal images were displayed as single optical sections (**b**). Fluorescence intensities quantified from three independent experiments were presented as mean ± s.d (**c**). Student's *t* test: * $P<0.05$; ** $P<0.01$.
