# Supplementary material for: abLIM1 constructs non-erythroid cortical actin networks to prevent mechanical tension-induced blebbing
Source: Cell Discov. 2018 Jul 24;4:42. doi: 10.1038/s41421-018-0040-3 (PMC6056535; doi:10.1038/s41421-018-0040-3)
Supplement: Supplementary file 5 — Readme file for supplementary information [file 41421_2018_40_MOESM5_ESM.docx]

The supplementary information contains four files, including:

1. One PDF file containing six Supplementary Figures.

2. Three MOV files containing Supplementary Videos 1-3, respectively.
